# Hypoxic adaptation theory of cancer

**DOI:** 10.3389/fcell.2026.1731939

**Published:** 2026-04-02

**Authors:** Edmund K. Kwan, Justin Park

**Affiliations:** 1 Department of Plastic and Reconstructive Surgery, New York Presbyterian Hospital, New York, NY, United States; 2 New York Presbyterian Hospital-Cornell Medical Center Weill-Cornell Medical College, New York City, NY, United States; 3 Rutgers New Jersey Medical School, Newark, NJ, United States

**Keywords:** epigenome, HIF-1α, hypoxia, hypoxic adaptation theory, pathological angiogenesis

## Abstract

For more than 70 years, the somatic mutation theory (SMT) has dominated cancer biology, conceptualizing carcinogenesis as the cumulative consequence of genetic mutations, However, expanding molecular and microenvironmental evidence reveals important limitations in this mutation-centric framework. The Hypoxic Adaptation Theory (HAT) reframes carcinogenesis not as a purely mutation-driven process, but as the maladaptive culmination of chronic cellular hypoxia. HAT integrates with SMT by situating mutagenesis within a microenvironmental and evolutionary context rather than opposing it, positioning sustained oxygen deprivation as a primary upstream driver of genomic instability and malignant transformation. Carcinogenic exposures—whether physical, chemical, or biological—not only induce direct DNA damage but also converge on a shared pathogenic pathway characterized by cellular injury, chronic inflammation, microvascular disruption, and impaired oxygen delivery. Persistent hypoxia stabilizes hypoxia-inducible factor (HIF), initiating metabolic reprogramming toward glycolysis, pathological angiogenesis, and enhancing cellular plasticity. These adaptive responses may drive phenotypic transitions from hyperplasia to metaplasia, dysplasia, and ultimately neoplasia. Concurrently, chronic hypoxia imposes significant epigenetic pressure, remodeling chromatin accessibility, suppressing DNA repair pathways, and reprogramming transcriptional networks that support survival under low-oxygen conditions. Although initially protective, prolonged HIF activation progressively destabilizes genomic integrity, fosters mutational retention, and reinforces oncogenic behavior. Importantly, HAT situates cancer within a broader continuum of hypoxia-driven chronic diseases, encompassing cardiovascular, metabolic, neurodegenerative, and inflammatory disorders. By shifting emphasis from random mutation to chronic hypoxic stress, HAT offers a unifying model of disease pathogenesis and identifies oxygen homeostasis as both a central biological vulnerability and a promising therapeutic target.

## Introduction

The somatic mutation theory of cancer, first introduced in 1914 by zoologist Theodor Boveri, remains the dominant paradigm of carcinogenesis. In his seminal work, Boveri postulated that cancer was fundamentally a unicellular phenomenon arising from chromosomal abnormalities (Soto and Sonnenschein, 2014). With major advances in molecular biology in the second half of the twentieth century—including the discovery of oncogenes and tumor suppressor genes—this framework was reinforced, promoting a reductionist view that directly linked genotype and phenotype (Sonnenschein and Soto, 2020).

While substantial evidence supports the presence of driver mutations in tumors, their role in carcinogenesis is often overstated (Martínez-Jiménez et al., 2020). Although numerous cancer-associated genes have been identified, some malignancies show no detectable mutations in nuclear DNA, and many mutated genes never progress to cancer. Moreover, a significant proportion of mutations appear only after malignant transformation, suggesting they may be consequences of the cancerous state rather than its primary cause (Seyfried et al., 2025).

As early as 1863, long before the molecular era, Rudolph Virchow proposed a connection between inflammation and cancer, asserting that “chronic irritation” underlies cancer development. Though largely speculative at the time, modern research has validated his insight: chronic inflammation is now recognized as a fundamental driver of both disease and tumorigenesis (Singh et al., 2019).

In 2000, Hanahan and Weinberg, articulated six hallmarks of cancer: sustaining proliferative signaling, evading growth suppressors, resisting cell death, enabling replicative immortality, inducing angiogenesis, and activating invasion and metastasis (Hanahan and Weinberg, 2000) —each underpinned by genomic instability. In 2011, they expanded this framework to include two additional hallmarks: metabolic reprogramming and immune evasion, underscoring the importance of an unstable microenvironment (Hanahan and Weinberg Robert, 2011).

While driver mutations can arise in both normoxic and hypoxic cells, and many non-hypermutated cancers exhibit conserved mutational spectra that do not require low oxygen to emerge, accumulating evidence suggests that unfavorable conditions—particularly chronic hypoxia—play a much greater role in cancer initiation than previously appreciated. Sustained oxygen deprivation imposes intense genomic and epigenomic pressure that can destabilize DNA integrity and, over time, facilitate the accumulation and retention of mutations. In this contexts, malignant transformation may represent a biological tradeoff: short-term cellular survival at the expense of long-term genomic stability. Hypoxia activates adaptive programs that promote metabolic flexibility and survival but simultaneously suppress DNA repair capacity, enhance phenotypic plasticity, and progressively erode genomic fidelity. These observations position chronic hypoxia as a potential upstream driver of carcinogenesis within, rather than outside, the broader mutation-based framework.

## Hypoxia and chronic inflammation

When a foreign invader or toxins enter the body—whether from trauma, microbial invasion, or a noxious substance—tissue damage occurs. The immune system responds with acute inflammation. Once the threat is neutralized, healing begins. However, if the offending agent is not fully eliminated, inflammation can persist and become chronic. Unlike acute inflammation, chronic inflammation is prolonged and often driven by unresolved infection or ongoing immune activation. Chronic inflammation is slow, long-term process that can last months to years. It is sustained by a persistent triggering agent that keeps the immune system activated. Common causes include.

1. Failure to eliminate the initial cause of acute inflammation.
2. Ongoing exposure to a low level of an irritant or foreign material that cannot be cleared.
3. Autoimmune disorders where the immune system mistakenly attacks healthy tissues.
4. Defects in inflammatory response that lead to persistent or recurrent inflammation.
5. Repeated episodes of acute inflammation.
6. Oxidative stress and mitochondrial dysfunction (Pahwa et al.,2023).

Chronic inflammation is a defense mechanism evolved to preserve cellular integrity. Without it, cells under persistent stress or irritation would be overwhelmed and fail. The body is compelled to react to virtually any sustained irritant—whether physical, chemical, or biological. Regardless of the source—be it toxins, viruses, or bacteria—the immune system responds in a similar way, aiming to contain the threat and restore tissue function.

During acute inflammation, vasodilation and increased vascular permeability cause redness, swelling, and enhanced blood flow, facilitating elimination of the offending agent. By contrast, chronic inflammation is characterized by reduced blood flow and impaired tissue perfusion. While it signals an unresolved problem, it also represents a survival mechanism, enabling cells to endure when immediate resolution is unattainable. Despite its long-term costs, chronic inflammation prioritizes survival in the body’s hierarchy of responses (Kiss, 2022).

Chronic inflammation drives profound structural and functional alterations across the vascular network, from large arteries to arterioles, capillaries, and venules. Immune cell infiltration and cellular debris can obstruct the blood flow, while endothelial injury increases vascular permeability and disrupts hemodynamic balance. Inflammatory cytokines induce vasoconstriction and capillary rarefaction and activated neutrophils release ROS that exacerbate endothelial damage (Pober and Sessa, 2014). Reduced endothelium-dependent nitric oxide (NO) bioavailability impairs vasodilation, further compromising flow. Concurrently, rising blood viscosity, diminished blood cell deformability, and leukocyte activation decrease fluidity. Fibrosis and extracellular matrix deposition replace compliant tissue with rigid, nonfunctional scar. Platelet hyperactivity adds to the risk of microvascular thrombosis, while pro-inflammatory mediators perpetuate clot formation and delay resolution. Compensatory angiogenesis, though initiated to restore oxygenation, paradoxically prolongs and intensifies the inflammatory cascade (Granger and Senchenkova, 2010; Kim and Lee, 2025; Afsar et al., 2018). Collectively, these vascular and rheological disturbances impair oxyhemoglobin transport, restrict perfusion, and culminate in tissue hypoxia.

## Hypoxia and hypoxia inducible factor (HIF)/angiogenesis

As oxygen levels drop, cells transition into survival mode. Mammalian cells depend on a built-in oxygen-sensing mechanism called hypoxia inducible factor (HIF). Under hypoxic conditions, HIF-1⍺ is stabilized in the cytoplasm, translocates to the nucleus, and binds to HIF-1β. This heterodimeric protein complex acts as a transcription factor, altering the expression of over 150 genes. As a result, energy metabolism shifts to glycolysis, glucose uptake increases, and vascular endothelial growth factor (VEGF) is activated, promoting angiogenesis (McGettrick and O’neill, 2020; Semenza, 2002).

The nature of angiogenesis is deeply shaped by the surrounding cellular environment. Although both physiological and pathological angiogenesis are initiated by hypoxia and accompanying oxygen gradients, their outcomes diverge sharply. In physiological contexts—such as fetal development, tissue growth, wound healing, endometrial proliferation during ovulation, and skeletal muscle adaptation during exercise—oxygen consumption transiently exceeds supply, producing localized hypoxia within an otherwise stable oxygen environment. This controlled hypoxic stimulus promotes the development of well-organized, functionally efficient vascular network, characterized by orderly branching of arterioles, capillaries, and venules (Dudley and Griffioen, 2023).

By contrast, chronic inflammation produces a profound and persistent oxygen deficit. The angiogenesis that follows is markedly abnormal: vessels are haphazard, dilated, and leaky, lacking the hierarchical architecture of normal vasculature. Despite their abundance, blood flow remains erratic, and tissue hypoxia persists. Microvascular disruption compromises the capillary-tissue interface essential for oxygen exchange, while the widened oxyhemoglobin diffusion distance further limits oxygen delivery (Guven et al., 2020; Roy and Secomb, 2020).

In cancer and other pathological states, angiogenesis is rampant yet profoundly dysfunctional. The resulting vascular networks are spatially disorganized, forming maze-like labyrinth characterized by tortuous architecture, irregular caliber, and even retrograde blood flow. Although these aberrant vessels fail to restore adequate tissue oxygenation, they nonetheless permit diffusive transport of nutrients. Cancer cells exploit this limitation by upregulating solute carrier (SLC) transporters—including GLUT1, SGLT, LAT1, MCTs and FATP—thereby facilitating the uptake of glucose, amino acids, fatty acids, and other metabolites, even against steep concentration gradients. This metabolic reprogramming sustains the heightened energetic and biosynthetic demands of tumor cells, enabling continued proliferation within chronically hypoxic environments (Zhao et al., 2015; Pliska and Szablewki, 2021). However, the chaotic vascular architecture severely disrupts normal hemodynamics, impairing the efficient transit of red blood cells and immune effector cells. The resulting hypoxic and immunosuppressive tumor microenvironment further compromises immune surveillance and function. Paradoxically, cancer cells thus remain metabolically well supplied yet persistently oxygen-deprived, while simultaneously shielded from immune detection—conditions that collectively foster tumor progression and malignancy (Adekola et al., 2012; Hu et al., 2022).

## Hypoxia and epigenome

Phenotypic alterations can arise in cells independently of DNA sequence changes through epigenetic mechanisms. These heritable modifications, established during development and differentiation, include DNA methylation, post-translational histone modifications, and microRNA-mediated regulation of gene expression. Epigenomic pathways play a central role in cancer initiation, particularly under adverse environmental conditions. Among these stressors, persistent hypoxia emerges as a dominant driver of epigenetic reprogramming. Chronic hypoxic exposure reshapes transcriptional landscapes through coordinated epigenetic regulation, effectively rewiring cellular programs to promote survival and adaptation. Over time, sustained reprogramming can impose long-lasting developmental constraints that predispose cells to malignant transformation (Almalki, 2025).

Hypoxia exerts many of its epigenetic effects through HIF-mediated modulation of hypoxia-responsive genes, primarily by altering chromatin structure and accessibility. This regulation is tightly linked to oxygen-dependent chromatin-modifying enzymes, including prolyl hydroxylases and multiple histone-modifying complexes. Under hypoxic conditions, histone methyltransferases and demethylases dynamically remodel histone methylation patterns, resulting in chromatin reorganization that either relaxes or condenses chromatin and thereby modulates transcriptional activity. Histone acetylation and deacetylation, mediated by histone acetyltransferases and histone deacetylases (HDACs), Further regulate transcriptional output. Histone acetylation generally promotes an open chromatin state conducive to gene expression, whereas HDAC activity reinforces chromatin condensation and transcriptional repression. HDAC-mediated silencing can suppress key regulatory genes such as TP53, VHL (Von-Hippel-Lindau), and RECK (reversion-inducing cysteine-rich protein with Kazal motifs), while concurrently enhancing HIF-1⍺ stability and amplifying hypoxia-adaptive signaling pathways (Kim et al., 2022).

In parallel, hypoxia profoundly reshapes DNA methylation landscapes across the genome. Through HIF-dependent modulation of Ten-Eleven Translocation (TET) dioxygenases and DNA methyltransferases (DNMTs), hypoxia can induce both global hypomethylation and locus-specific hypermethylation at CpG sites. Hypoxic exposure has been associated with increased expression and activity of DNMT1 and DNMT3B, promoting maintenance and *de novo* methylation, respectively, and leading to transcriptional repression of targeted genes. These methylation alterations frequently occur at promoter-associated CpG islands and regulatory regions, where hypermethylation can silence tumor suppressor genes, while global hypomethylation may contribute to genomic instability and aberrant activation of oncogenic pathways. Because TET enzymes are oxygen- and ⍺-ketoglutarate-dependent dioxygenases, reduced oxygen availability can directly impair their catalytic activity, further skewing the balance toward methylation accumulation (D’Anna et al., 2020; Verdikt and Thienpont, 2024).

Hypoxia also regulates gene expression at the post-transcriptional level through microRNAs, particularly a subset known as hypoxamirs. These noncoding RNAs fine-tune hypoxic responses by targeting mRNAs involved in metabolism, angiogenesis, apoptosis, and cell-cycle control, further reinforcing adaptive cellular phenotypes under low-oxygen conditions (Egea, 2025).

At the chromatin level, hypoxia induces targeted remodeling through histone-modifying enzymes that regulate methylation, acetylation, phosphorylation, ubiquitination, propionylation, crotonylation. Lysine-specific demethylases 1 (LSD1/KDM1A) removes methyl groups from histone H3 lysine 4 (H3K4me1/2), typically reducing chromatin accessibility and repressing transcription. The histone methyltransferase G9a (EHMT2) becomes stabilized under hypoxia, increasing H3K9 mono- and demethylation (H3K9me1/2), which promotes transcriptional silencing of specific genes, including *HHEX, GATA2,* and *ARNTL*. Reptin (RUVBL2), when methylated, functions as a transcriptional corepressor, modulating hypoxia-responsive pathways. HDACs further reinforce repression by inducing H3K9 deacetylation and chromatin condensation. Members of the Jumonji C (JmjC) domain-containing demethylase family, such as KDM5A (JARID1A), are oxygen-dependent enzymes whose catalytic activity diminishes under hypoxia. Reduced KDM5A activity alters H3K4 trimethylation (H3K4me3) dynamics, reshaping transcriptional regulation at hypoxia-sensitive loci. Notably, hypoxia induces selective, locus-specific histone methylation changes rather than uniform global repression (Kim et al., 2022). In some cases, transient hypoxic episodes permit epigenetic alterations to persist following reoxygenation through the establishment of “hypoxic memory”. This form of epigenetic memory is characterized by the retention of bivalent chromatin domains—marked by the coexistence of H3K4me3 (an active mark) and H3K27me3 (repressive mark)—at specific genomic loci. Such bivalency maintains genes in a poised but transcriptionally restrained state, preventing full restoration of baseline gene expression after oxygen levels normalize. The result is durable gene silencing or altered transcriptional responsiveness, thereby stabilizing hypoxia-induced phenotypic reprogramming beyond the initial stimulus (Prickaerts et al., 2016; Kwoun et al., 2025).

Collectively, the epigenome functions as regulatory “software” governing genomic expression, and epigenetic alterations can phenocopy genetic mutations by silencing tumor suppressor genes and activating oncogenes. Chronic hypoxia, acting through sustained HIF activation and oxygen-sensitive chromatin-modifying enzymes, represents a major source of epigenetic pressure that reshapes gene expression landscapes, redefines cellular identity, survival adaptation, and contributes to malignant phenotypes (Cricchi et al., 2025).

## Hypoxia and energy metabolism

Energy production is fundamental to all living organisms, particularly in mammals, where the scale and complexity of cellular networks demand high energy output. Under normoxic conditions, mammalian cells rely predominantly on oxidative phosphorylation (OxPhos)—an oxygen-dependent process that generates majority of cellular ATP. During hypoxia, OXPHOS efficiency declines, leading to reduced ATP production. To compensate, cells increase reliance on glycolysis, an oxygen-independent pathway that generates ATP at substantially lower efficiency (Rigoulet et al., 2020).

In most mammals, bioenergetic demand remains relatively constant despite fluctuations in oxygen availability. Under hypoxic stress, cells undergo epigenetic and transcriptional reprogramming that enhances glucose uptake and glycolytic flux. Although this metabolic shift sustains ATP production, it requires increased glucose consumption and promotes accumulation of metabolic byproducts, contributing to acidosis, oxidative stress, and inflammatory signaling (Sun et al., 2025). By contrast naked mole-rat (NMR) exhibits a remarkable capacity to downregulate metabolic demand in proportion to oxygen availability—an evolutionary adaptation to its chronically hypoxic subterranean environment. During sustained low-oxygen exposure, NMR cells enter a hypometabolic state, suppressing nonessential cellular processes and reducing overall ATP demand rather than compensating through glycolytic overdrive. This metabolic restraint limits the accumulation of toxic metabolites and preserves cellular integrity. In addition, NMRs display attenuated inflammatory responses to chemical and environmental stressors, in sharp contrast to the self-amplifying inflammatory cycles observed in hypoxia-sensitive species (Kadamani et al., 2024). Together with the production of ultra-high-molecular-weight hyaluronic acid, stringent cell-cycle control, and highly efficient DNA repair mechanisms, these adaptations contribute to their extraordinary longevity—up to tenfold longer than similarly sized rodents (Seluanov et al., 2018).

Notably, NMR’s paradoxically exhibit significantly higher basal expression of HIF-1⍺ and VEGF compared with hypoxia-sensitive mice. Unlike mice, NMRs stabilize HIF-1⍺ even under normoxic conditions by limiting its ubiquitination and proteasomal degradation. However, despite elevated HIF-1⍺ levels, downstream hypoxia-responsive target genes remain largely downregulated. This partial uncoupling of HIF stabilization from full transcriptional activation appears to buffer NMRs from the deleterious consequences of chronic HIF signaling, including uncontrolled angiogenesis and metabolic dysregulation (Faulkes et al., 2024).

Furthermore, hypoxia readily induces apoptosis in mouse cells but not in NMR cells, underscoring their exceptional resilience to oxygen deprivation (Xiao et al., 2017). This resistance is supported by enhanced DNA damage surveillance and repair capacity. NMRs exhibit adaptations in innate immune DNA-sensing pathways, including cyclic GMP-AMP synthase (cGAS) signaling, which facilitate efficient clearance of damaged DNA while limiting inflammatory activation (Chen et al., 2025). In addition, recent comparative study indicates that NMR-specific regulation of chromatin-associated proteins contributes to their hypoxia tolerance, for example, overexpression of histone H1.2 during oxygen deprivation has been associated with early stabilization of HIF while concurrently suppressing cancer cell migration and invasion. This suggests a distinctive modulation of hypoxia signaling that preserves adaptive responses without triggering pro-oncogenic programs. Collectively, these mechanisms enable NMRs to tolerate chronic hypoxia without engaging the maladaptive metabolic, inflammatory, and angiogenic cascades that predispose other mammals to disease and malignancy (Du et al., 2024).

## Hypoxia and chronic diseases

**FIGURE 1:**
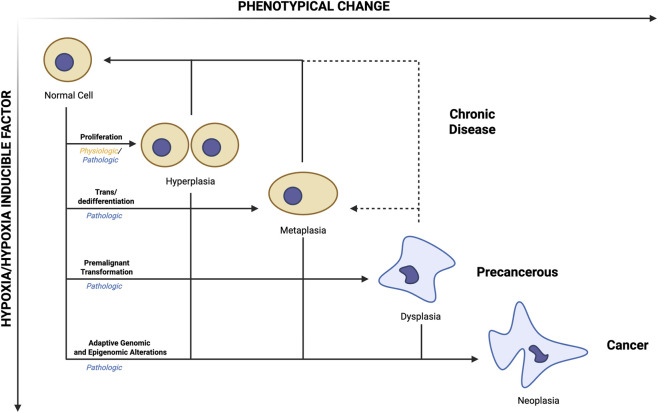
Schematic representation of cellular plasticity across a gradient of hypoxic stress and HIF activation. As hypoxia intensifies, adaptive cellular responses typically progress from hyperplasia to metaplasia, dysplasia, and ultimately neoplasia. In certain contexts, severe or prolonged hypoxia may drive direct transition to dysplasia or neoplasia, bypassing intermediate stages. Hyperplastic, metaplastic, and early dysplastic states are potentially reversible upon restoration of oxygen homeostasis, whereas late dysplastic and neoplastic transformations are considered irreversible. Hyperplasia and metaplasia correspond to chronic disease states, while dysplasia and neoplasia represent precancerous and malignant conditions. Conceptually, chronic hypoxia imposes adaptive genomic and epigenomic pressures that drive malignant transformation.

When hypoxia persists, cells mobilize adaptive survival mechanisms mediated by HIF signaling. These adaptations often unfold along a continuum of structural and phenotypic changes, beginning with hyperplasia, advancing to metaplasia and dysplasia, and ultimately neoplasia (Figure 1) (Stancu et al., 2007). Disease may therefore be conceptualized as a spectrum, ranging from homeostasis to malignancy. Along this continuum, cells experience sustained selective pressures, with the genotype expressing phenotypes best suited to the prevailing microenvironment. Among these pressures, oxygen availability is a dominant determinant, exerting broad control over cellular behavior, gene expression, and tissue architecture. Acting as the central oxygen sensor, HIF regulates the expression of more than 2% of human genes and functions as a molecular messenger, orchestrating transcriptional, metabolic, and structural adaptation to hypoxic stress (Hu et al., 2023).

Chronic inflammation is a common precursor to myriad of acquired chronic diseases. Cardiovascular disease, metabolic disorders, neurodegenerative diseases, and cancer all share a unifying feature: cellular hypoxia plays a central role in both the initiation and progression of these disease processes (Hypoxic Adaptation Theory of disease) (Kwan et al., 2023).

### Cardiovascular disease (CVD)

CVD encompasses conditions such as hypertension, heart disease, peripheral vascular disease, stroke, and myocardial infarction. Its pathology often begins with vascular inflammation, which initiates endothelial dysfunction, lipid deposition, plaque formation, ultimately leading to fibrosis and calcification. These structural changes narrow vessel lumen, reduce vascular elasticity, and impair blood flow—significantly increasing the risk of adverse cardiovascular events.

Persistent inflammation thickens the intima and damages the vasa vasorum, producing localized hypoxia within the vascular smooth muscle cells (VSMCs). In response, VSMCs undergo hyperplastic transformation, contributing to vessel wall thickening and early atherosclerotic change. With continued insult, metaplastic changes emerge, marking the progression of the disease. Hypoxia and HIF is central to this process, driving VSMC plasticity and shifting cells from a contractile, quiescent phenotype to a synthetic, proliferative, and migratory state. HIF orchestrates much of this adaptive response while inducing pathological angiogenesis that exacerbates vascular remodeling and accelerates disease progression (Sorokin et al., 2020).

### Metabolic disease

Hyperglycemia induces inflammation and cellular hypoxia due to nutrient overload and elevated inflammatory mediators. Pancreatic beta cells are highly metabolic and rely on a steady oxygen supply. Persistent hypoxia severely impairs their function. HIF activation and pathological angiogenesis contribute significantly to beta cell damage. In response, the body attempts to regenerate beta cells through phenotypic changes in neighboring cells—such as alpha, ductal, acinar cells, and even hepatocytes. Over time, this compensatory mechanism leads to cellular exhaustion, beta cell burnout, and the eventual onset of diabetes (Liu et al., 2020).

### Rheumatoid arthritis (RA) and osteoarthritis (OA)

OA is a degenerative joint disease, while RA is an autoimmune disorder primarily targeting the synovial joints. Despite their distinct origins, both share a common inflammatory and hypoxic microenvironment. In RA and OA, disease activity centers within the synovium, where persistent hypoxia and HIF pathway activation drive inflammatory signaling, cellular plasticity, and pathological angiogenesis. The sustained oxygen deficit perpetuates synovial inflammation, disrupts cartilage homeostasis, and leads to progressive cartilage degradation and joint destruction (Du et al., 2024; Cutolo et al., 2025; Varela-Eirin et al., 2018).

### Age-related macular degeneration (AMD)

AMD is the leading cause of vision loss in the elderly. It results from progressive damage to the macula—the central part of the retina responsible for sharp vision. The retina contains some of the most metabolically active cells in the body, requiring a constant and abundant oxygen supply to maintain function (Yu et al., 2011). In AMD, chronic inflammation plays a central role, leading to retinal hypoxia and triggering pathological angiogenesis. Prolonged hypoxia unleashes an adaptive response, activating HIF, upregulating VEGF, and shifting metabolism toward glycolysis. However, HIF-induced angiogenesis results in structurally abnormal, fragile blood vessels that are prone to leakage. Retinal cells are trapped in a vicious cycle of fluctuating hypoxia and pathological vessel growth, with oxygen levels progressively declining over time. As the disease advances, the chronic hypoxic state ultimately results in the destruction of retinal cells (Lee et al., 2024).

### Neurodegenerative diseases

Alzheimer’s disease (AD) is the most common form of dementia, primarily affecting the hippocampus and leading to progressive memory loss. While its exact pathology remains uncertain, it shares key features with other neurodegenerative diseases (Parkinson’s, ALS, MS, Huntington’s disease)—namely, neuroinflammation, diminished cerebral blood flow, chronic hypoxia, and neuronal cell death.

Microglia are resident macrophages in the brain, while astrocytes—another type of glial cell—play a critical role in maintaining neuronal health and regulating the synaptic environment. In response to injury and insult, neuroinflammation and hypoxia follow, activating the HIF pathway (Zhang et al., 2023). During this process, both microglia and astrocytes may undergo phenotypic changes that have detrimental effects, contributing to the development of neurodegenerative diseases. Over time, hallmark features such as amyloid plaques and tau tangles appear. The risk of AD is notably higher in individuals with cardiovascular disease, as reduced capillary density and microvascular injury impair cerebral perfusion, further exacerbating neuronal damage (Wendimu and Hooks, 2022; Kim et al., 2024).

## Hypoxia and reactive oxygen species (ROS)

Reactive oxygen species (ROS) are highly unstable, oxygen-containing molecules produced as byproducts of cellular metabolism. They include hydroxyl radicals, superoxide, hydroperoxides, and singlet oxygen. While ROS can serve beneficial roles as signaling molecules, excessive accumulation becomes destructive, inflicting oxidative damage to DNA, proteins, and cell membranes. This oxidative stress contributes to genetic instability and can trigger apoptosis (Bardaweel et al., 2018). In mammalian cells, ROS are produced across multiple subcellular compartments, including mitochondria, endoplasmic reticulum, peroxisomes, lysosomes, cytoplasm, and the plasma membrane. Under normal conditions, redox balance is maintained by antioxidant systems such as superoxide dismutase, catalase, and glutathione peroxidase. During sustained stress or chronic hypoxia, these protective mechanisms become overwhelmed, tipping the balance toward oxidative injury and disease progression (Jena et al., 2023).

Paradoxically, hypoxia frequently enhances ROS production. While acute hypoxia may transiently reduce ROS generation, chronic hypoxia amplifies ROS output despite limited oxygen availability. At the mitochondrial level. Hypoxia compromises electron transfer efficiency within the electron transport chain, particularly at complex IV, impairing radical neutralization. Electrons leak upstream and react with residual oxygen, resulting in excessive mitochondrial ROS generation—an effect that is further exacerbated during hypoxia-reoxygenation cycles (Tafani et al., 2016).

Hypoxia-induced ROS play a central role in driving genomic instability. Elevated ROS levels induce DNA strand breaks, oxidative base lesions such as eight-oxo-guanine, and replication stress characterized by stalled or collapsed replication forks. These effects are especially pronounced during reoxygenation, when sudden oxygen influx amplifies oxidative damage beyond the capacity of DNA repair pathways (Wang et al., 2025).

ROS and hypoxia are also critical regulators of epithelial-mesenchymal transition (EMT). A process that enhances cellular motility, invasion, and stem-like behavior. ROS activate key signaling pathways—including transforming growth factor-β (TGF-β), NF-κB (nuclear factor-κB), and GSK (glycogen synthase kinase) signaling—thereby reinforcing transcriptional programs associated with invasion and metastasis (Mendoza et al., 2025).

The HIF pathway is tightly coupled to ROS signaling. Mitochondrial ROS act as secondary messengers that inhibit prolyl hydroxylases (PHDs), stabilizing HIF-1⍺ and promoting activation of hypoxia-responsive genes. In cancer, elevated ROS levels—driven by altered metabolism, mitochondrial dysfunction, and impaired antioxidant defenses—further amplify HIF signaling. This feed-forward loop promotes pathological angiogenesis, metabolic reprogramming, and immune evasion (Snyder and Chandel, 2009).

Beyond genomic instability, hypoxia-induced ROS contribute to tumor immune remodeling. HIF-dependent pathway facilitates recruitment of immunosuppressive cell populations, including tumor-associated macrophages (TAM) and myeloid-derived suppressor cells, while reprogramming T-cell function and remodeling the extracellular matrix to create physical and immunologic barriers to immune surveillance. Collectively, hypoxia-ROS-HIF interactions establish a self-reinforcing microenvironment that drives malignancy (Nakamura and Takada, 2021).

## Hypoxia and Warburg effect (WE)

Otto Warburg first observed that cancer cells preferentially rely on glycolysis rather than oxidative phosphorylation (OxPhos), even in the presence of adequate oxygen—a phenomenon now known as the “Warburg Effect” (WE). This metabolic reprogramming reflects coordinated changes in gene expression and mitochondrial function that prioritize rapid ATP production and biomass accumulation over metabolic efficiency (Burns and Manda, 2017).

Although strongly associated with cancer, the WE is not unique to malignancy. It also occurs in rapidly proliferating cells under both physiological and pathological conditions, where HIF signaling drives a transcriptional shift toward glycolytic metabolism (Abdel-Haleem et al., 2017). Mechanistically, HIF-1⍺ suppresses mitochondrial respiration by inducing pyruvate dehydrogenase kinase-1 (PDK1), which inhibits pyruvate dehydrogenase and blocks conversion of pyruvate to acetyl-CoA. As a result, pyruvate is diverted away from the tricarboxylic acid cycle and instead converted to lactate, regenerating NAD^+^ and sustaining high glycolytic flux (Tang et al., 2025).

In physiological settings—such as immune activation, tissue repair, and high-intensity exercise—the WE supports biosynthetic demands and enables cells to maintain function during transient stress or oxygen limitation. This metabolic flexibility allows for rapid adaptation without long-term pathological consequences.

In contrast, in pathological states, persistent hypoxia stabilizes HIF-1⍺, driving sustained epigenetic and transcriptional activation of genes involved in glycolysis, survival, proliferation, and angiogenesis. Although glycolysis yields less ATP per glucose molecule than OxPhos, it produces ATP up to tenfold faster—an advantage in environments with fluctuating or limited oxygen availability (Kukurugya et al., 2024). Under chronic hypoxia, this shift ensures a continuous energy supply while diverting glucose intermediates into anabolic pathways that fuel rapid cell growth. In cancer, this metabolic state becomes fixed, with cells relying predominantly on glycolysis even when oxygen is sufficient (Vaupel and Multhoff, 2021).

HIF-1⍺ functions as a master regulator of glycolytic gene expression and a central mediator of the Warburg phenotype. Enhanced glycolysis generates moderate levels of ROS, which act as signaling molecules that promote cell proliferation and survival. Simultaneously, hypoxia induced alterations in mitochondrial redox potential reshape ROS dynamics, linking metabolic flux to transcriptional regulation (Zhong et al., 2022).

Elevated glycolytic activity also increases NADPH production, supporting antioxidant systems such as glutathione regeneration and buffering ROS generated under hypoxic conditions. This redox control protects cancer cells from oxidative damage and apoptosis, reinforcing survival in oxygen-deprived environments. Collectively, these redox-dependent signals influence chromatin remodeling through effects on histone acetylation, thereby modulating DNA repair capacity and gene expression programs (Pramono et al., 2020).

Beyond intracellular effects, the WE profoundly reshapes the tumor microenvironment. Cancer cells upregulate glucose transporters such as GLUT1 to meet metabolic demand, while excessive lactate production acidifies the extracellular space. This acidic milieu suppresses immune surveillance, promotes extracellular matrix remodeling, and facilitates invasion and metastasis (Liberti and Locasale, 2016; Barba et al., 2024).

## Hypoxia and carcinogens

Human carcinogens have been categorized according to ten defining characteristics that collectively capture carcinogenic potential: (1) electrophilicity or metabolic activation; (2) genotoxicity; (3) disruption of DNA repair; (4) induction of epigenetic alterations or genomic instability; (5) generation of oxidative stress; (6) promotion of chronic inflammation; (7) immunosuppression; (8) activation of receptor-mediated signaling pathways; (9) induction of cellular immortalization, and (10) dysregulation of cell proliferation, cell death, and angiogenesis (Smith et al., 2020). This framework underscores that carcinogenesis is not solely the consequence of direct DNA damage but reflects a broader spectrum of biological perturbation.

Notably, many of the same agents that drive chronic inflammatory and degenerative diseases also function as carcinogens. More than 8,000 compounds have been classified as carcinogenic, including 1,400 confirmed or suspected human carcinogens. These agents activate pro-inflammatory, pro-angiogenic, pro-tumorigenic mediators—including TNF-⍺, IL-6, and NF-κB, and multiple proto-oncogenes. Among these, NF-κB plays a central role in sustaining chronic inflammation, while many carcinogens simultaneously impair the resolution through immunosuppressive effects (Nishida and Andoh, 2025; Korbecki et al., 2021). In parallel, carcinogenic exposures frequently induce cyclooxygenase-2 (COX-2) expression, further amplifying inflammation, angiogenesis, and tumor promotion (Gandhi et al., 2017).

Although many carcinogens exert direct genotoxic effects—through base modifications, strand breaks, adduct formation, cross-linking, and oxidative lesions that can generate DNA damage—cells possess robust and highly coordinated repair systems to preserve genomic integrity. These include mismatch repair (MMR), nucleotide excision repair (NER). Base excision repair (BER), homologous recombinant (HR), non-homologous end-joining (NHEJ), and translesion synthesis (TLS). Each pathway operates at defined stages of the cell cycle to detect and resolve DNA damage prior to completion of replication and transcription, thereby maintaining genomic stability (Barnes et al., 2018). Genotoxicity alone, however, is often insufficient to initiate malignant transformation. When DNA repair pathways remain intact and cell-cycle checkpoints are functional, most lesions are corrected before replication fixation occurs, preventing mutation. Thus, the oncogenic potential of carcinogens cannot be attributed solely to their capacity to induce DNA damage.

**FIGURE 2:**
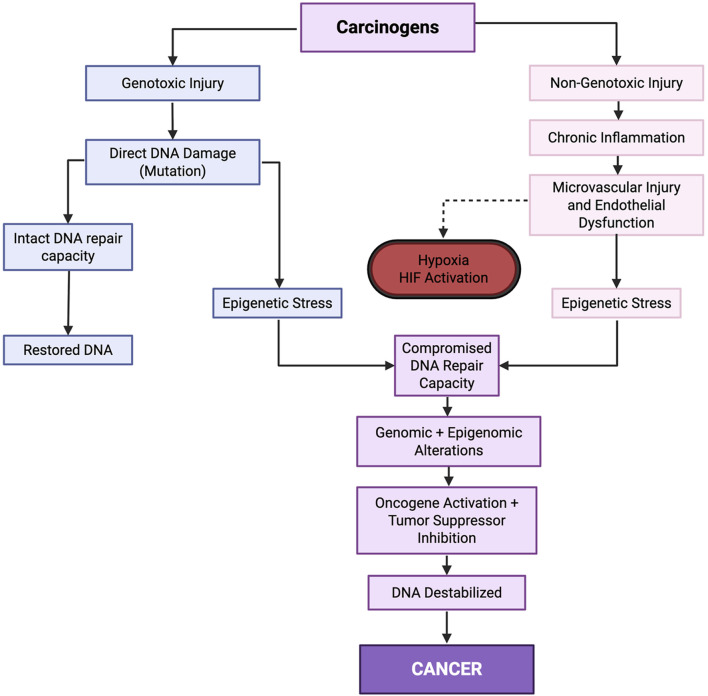
Carcinogens induce both genotoxic and non-genotoxic injuries through distinct mechanisms; however, these divergent pathways ultimately converge on chronic hypoxia and HIF activation. Over time, this maladaptive response suppresses DNA repair, induces genomic and epigenomic alterations, activates oncogenic programs, inhibits tumor suppressors, erodes genomic stability, and drives malignant transformation.

Importantly, carcinogens also exert indirect, non-genotoxic effects that profoundly alter cellular physiology. Many precipitates chronic inflammation, microvascular injury, and tissue hypoxia, leading to sustained generation of ROS and oxidative stress (Fishbein et al., 2020). These processes amplify inflammatory signaling cascades, impair DNA repair capacity, destabilize genomic maintenance mechanism, and remodel the epigenetic landscape. Hypoxia, in particular, disrupts oxygen-dependent DNA repair enzymes and chromatin modifying complexes, further compromising repair fidelity and altering gene expression programs. Together, these mechanisms establish a self-perpetuating cycle of cellular injury, maladaptive repair, and phenotypic reprogramming (Kaplan and Glazer, 2019; Lee et al., 2013). Carcinogens therefore promote mutagenesis not only through direct DNA damage, but also through hypoxia-mediated suppression of repair pathways, epigenetic dysregulation, and microenvironmental remodeling (Figure 2). Even within the framework of SMT, carcinogenic exposures are recognized as powerful drivers of genomic instability that influence both cancer initiation and progression. Carcinogens can be broadly classified into physical, chemical, and biological categories.

*Physical carcinogens* include ionizing radiation and ultraviolet (UV) rays. UV exposure induces DNA photoproducts and oxidative damage that distort DNA structure; with chronic exposure, it also provokes sustained inflammation, which is strongly associated with skin carcinogenesis. Ionizing radiation damages DNA through direct bond breakage and ROS-mediated oxidative injury. In addition, it injures the vasculature—causing endothelial damage, vasculitis, and increased vascular permeability, particularly within microvascular and medium-sized vessels. These injuries perpetuate chronic inflammation and tissue hypoxia and are linked to hematologic malignancies, such as leukemia, as well as multiple solid tumors, through mechanisms that closely resemble those triggered by chemical and biological carcinogens.

*Chemical carcinogens*—including asbestos, tobacco smoke, alcohol, aflatoxin, arsenic, and related compounds—accumulate in target tissues where they induce direct DNA damage through adduct formation, strand breaks, and oxidative stress, while simultaneously promoting persistent inflammation. Asbestos causes chronic injury to the lung parenchyma and pleura, markedly increasing the risk of mesothelioma and lung cancer. Tobacco smoke induces widespread endothelial dysfunction, driving systemic inflammation and elevating cancer risk not only in the lungs but across multiple organ systems.

*Biological carcinogens* include oncogenic viruses, bacteria, and parasites that generate oxidative stress and DNA base damage. While acute infections may be cleared, persistent infections sustain chronic inflammation and tissue injury. Hepatitis virus can remain within the hepatocytes, producing prolonged inflammatory damage that progresses through fibrosis and cirrhosis to hepatocellular carcinoma. Human papilloma virus (HPV) induces chronic cervical inflammation, increasing the risk of cervical cancer. *Helicobacter pylori* disrupt the gastric lining, leading to chronic gastritis and a heightened risk of gastric adenocarcinoma (Blackadar, 2016; Fernandes et al., 2015).

Regardless of their diverse origins—chemical, physical, infectious, or environmental—many carcinogenic exposures converge on a sustained inflammatory cascade that disrupts tissue perfusion and impairs oxygen delivery. The resulting hypoxic microenvironment fosters both the acquisition and maintenance of the cancer stem cell (CSC) phenotype, in part through the induction of epithelial-mesenchymal transition (EMT). Within the niches, epigenetic reprogramming reshapes gene expression profiles, selectively favoring the survival and expansion of cells with stem-like properties. These CSCs possess self-renewal capacity, heightened resistance to chemotherapy and radiotherapy, and the ability to initiate and sustain tumor growth (Pramono et al., 2020; Liberti and Locasale, 2016). Cellular hypoxia stabilizes HIF, which imposes epigenetic pressure by remodeling chromatin architecture and activating transcriptional programs that support metabolic adaptation, cellular plasticity, and survival. Concurrently, hypoxia compromises DNA repair pathways impairing the repair fidelity and increasing genomic instability. This reduced repair capacity not only accelerates mutational accumulation but also facilitates phenotypic diversification. Together, hypoxia-driven epigenetic reprogramming and defective DNA repair reinforce malignant evolution (Liu et al., 2025).

## Hypoxic adaptation theory (HAT)

Oxygen plays a pivotal role in embryonic development, regulating stem cell differentiation organogenesis, vasculogenesis, and morphogenesis. During early fetal development, relatively low oxygen tension helps preserve pluripotency, promotes proliferation, and maintains the undifferentiated state (Simon and Keith, 2008). In mature cells, oxygen exerts functions that extend beyond its role as the terminal electron acceptor in the mitochondrial oxidative phosphorylation; It also modulates intracellular signaling pathways (Zhu and Bunn, 2001), transcriptional programs (Semenza, 1996), immune responses, and apoptotic regulation. Physiological oxygen tension at the cellular level typically ranges from approximately 2%–10%, depending on tissue type and metabolic demand (Tsiapalis et al., 2019). Highly metabolically active tissues are particularly sensitive to reductions in oxygen availability, whereas certain cell populations—most notably stem and progenitor cells—reside and function optimally within a hypoxic niche. Chronic hypoxia is often experimentally defined as oxygen concentrations between ∼0.1 and 3% sustained for 16–72 h or longer, although both the magnitude and duration of hypoxia are highly tissue- and context dependent (Camuzi et al., 2019). In response to oxygen deprivation, some cells undergo apoptosis, whereas others engage adaptive phenotypic reprogramming. These divergent outcomes reflect cell-specific thresholds that govern pro-survival and pro-death signaling pathways. The ultimate cellular response is determined by a dynamic interplay between intrinsic factors—such as metabolic state, mitochondrial capacity, and epigenetic configuration—and extrinsic cues, including oxygen tension, nutrient availability, inflammatory mediators, and growth factor signaling. Stochastic variability further influences these outcomes. Collectively, these regulatory mechanisms ensure selective elimination of vulnerable cells while preserving tissue integrity and homeostasis (Flushberg and Sorger, 2015).

While mutations are often credited with activating proto-oncogenes, epigenetic stressors—particularly hypoxia and HIF signaling—can independently drive their expression. Chronic hypoxia imposes profound selective pressure on cells, acting through DNA methylation, histone modifications, and microRNA regulation to remodel chromatin architecture, reprogram transcriptional networks, and progressively undermine genomic stability (Tsai and Wu, 2014). Under hypoxic conditions, oncogenic pathways such as KRAS (Kirsten rat sarcoma virus), and MET (mesenchymal-epithelial transition factor) may become upregulated, promoting VEGF activation, pathological angiogenesis, resistance to apoptosis, enhanced invasiveness, and maintenance of cancer stem-like properties (Zeng et al., 2010; Pennacchietti et al., 2003). Concurrently, hypoxia represses key tumor suppressors—including p53, VHL, and RECK—through mechanisms involving increased histone deacetylase (HDAC) activity and HIF-dependent transcriptional control (Zhang et al., 2021; Lee et al., 2010). Although low oxygen environments commonly induce cell-cycle arrest, in certain physiological contexts—such as stem cell niches and tissue repair—hypoxia supports tightly regulated self-renewal and proliferation. In cancer, however, this regulatory balance is subverted. Hypoxia downregulates minichromosome maintenance (MCM) proteins, impairing replication licensing and checkpoint control, disrupting contact inhibition, and facilitating HIF-1-driven cell-cycle progression. This erosion of replication fidelity and checkpoint restraint promotes uncontrolled proliferation, a defining hallmark of malignancy. In contrast, restoration of adequate oxygen often attenuates these malignant behaviors (Muz et al., 2015; Yadav and Polasek-Sedlackova, 2024; Hubbi and Semenza, 2015). Collectively, these coordinated alterations—oncogene activation, tumor suppressor repression, and dysregulation of cell-cycle control—illustrate how HIF-1 orchestrates both genetic and epigenetic reprogramming during cancer initiation and progression. Substantial evidence indicates that many genetic alterations observed in tumors are closely linked to HIF-1-dependent transcriptional activity, underscoring its central role as a master regulator of oncogenesis (Adamaki et al., 2012).

Environmental stressors—particularly chronic hypoxia—can increase DNA replication errors and promote genomic instability. Under physiological conditions, an elaborate and highly coordinated network of DNA repair pathways and cell-cycle checkpoints preserves genomic integrity. BER corrects small base lesions arising from depurination, deamination, alkylation, and oxidative damage. MMR resolves base-base mismatches and insertion-deletion loops generated during replication. NER removes bulky, helix-distorting lesions, such as those induced by ultraviolet radiation. DSBs and DNA interstrand crosslinks are repaired primarily through HR and NHEJ, with HR providing high-fidelity repair using a sister chromatid template (Chen et al., 2024). Replication stress activates checkpoint pathways governed by key regulatory kinases including PLK1, CHK1/CHK2, and WEE1, which coordinate cell-cycle arrest, DNA repair, or apoptosis to prevent propagation of damaged DNA. Under sustained hypoxic stress, coordinated repair fidelity can become compromised. Downregulation of repair pathways and checkpoint adaptation may permit damaged DNA to proceed through replication, facilitating the retention and clonal dissemination of mutations (Hasvold et al., 2016; Verma et al., 2019). Chronic hypoxia suppresses multiple DNA repair mechanisms, increases replication fork stalling, promotes accumulation of DSB, and alters post-translational modifications of DNA repair proteins. Moreover, reoxygenation following prolonged hypoxia can further intensify replication stress through bursts of ROS-mediated damage (Tang et al., 2021).

BRCA1 and BRCA2 are central to maintaining genomic stability by orchestrating high-fidelity HR-mediated repair of DSBs. Although germline BRCA mutations markedly increase cancer risk by compromising DNA repair, redox homeostasis, chromatin remodeling, and transcriptional regulation, such mutations alone are not sufficient to initiate malignancy. Rather, chronic environmental stress—particularly sustained hypoxia with persistent HIF signaling—can suppress HR and shifts DSB repair toward error-prone NHEJ (Gorodetska et al., 2019; Altwerger et al., 2023). Importantly, 90%–95% of breast cancers arise sporadically in the absence of inherited BRCA mutations. Prolonged hypoxia can epigenetically silence BRCA1 promoter through repressive histone modifications, functionally mimicking BRCA deficiency and reinforcing genomic instability (Lu et al., 2011).

**FIGURE 3:**
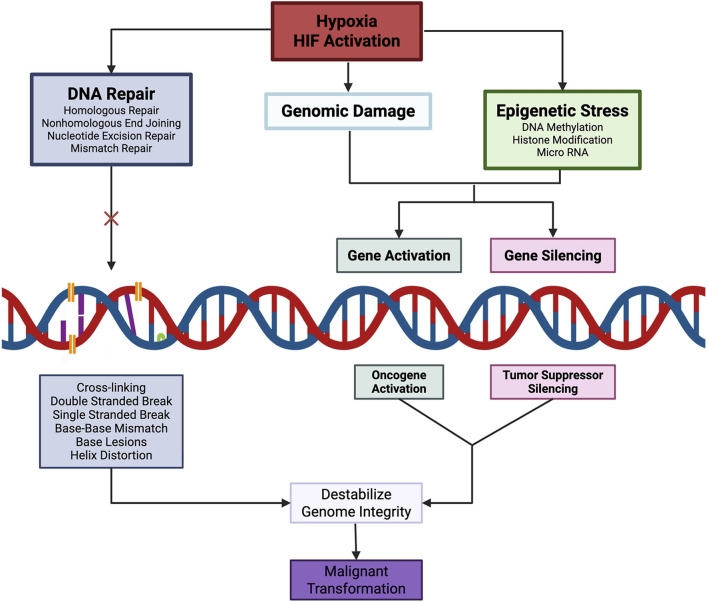
Chronic hypoxia and sustained HIF activation induce genomic damage and epigenetic stress, promoting oncogene expression while concurrently repressing tumor suppressor genes and impairing DNA repair pathways. Collectively, these alterations destabilize genomic integrity and establish a permissive cellular landscape for malignant transformation.

Through coordinated transcriptional, translational, post-translational, and epigenetic mechanisms, hypoxia activates oncogenic programs, inhibits tumor suppressor function, and progressively erodes DNA repair fidelity. These convergent effects destabilize the genome and establish a permissive landscape for malignant transformation (Figure 3).

Emerging experimental evidence, published in *Nature,* demonstrates that driver mutations may not be strictly required for tumor initiation. Transient epigenetic disruption—such as transcriptional silencing mediated by Polycomb group (PcG) proteins—has been shown to initiate tumorigenesis in the absence of canonical driver mutations. Using a *Drosophila* model, the authors showed that reversible depletion of PcG proteins was sufficient to induce cancer, and notably, malignancy persisted even after restoration of normal epigenetic regulation, indicating that a temporary epigenetic insult can irreversibly reprogram cellular identity and behavior (Parreno et al., 2024).

We therefore posit that chronic hypoxia, through sustained activation of HIF signaling, frequently precedes and facilitates the accumulation and retention of genetic mutations. Prolonged oxygen deprivation compromises cellular mechanisms that maintain genomic integrity, resulting in progressive genomic instability. Simultaneously, hypoxia activates HIF-dependent programs that drive sequential phenotypic transitions—progressing from hyperplasia to metaplasia and dysplasia, and ultimately culminating in malignant phenotype (Capp et al., 2024). Under persistent environmental stress, retention of mutations may confer selective survival advantages by enabling cells to bypass DNA repair pathways and cell-cycle checkpoint controls (Khouzam et al., 2023). Within this paradigm, carcinogenesis is re-envisioned not as a purely stochastic process driven solely by random mutations, but as an adaptive survival strategy mediated through coordinated genomic and epigenomic reprogramming in response to untenable environmental conditions.

## Testable predictions and experimental approaches

The Hypoxic Adaptation Theory (HAT) generates experimentally testable predictions that distinguish it from mutation-centric models of carcinogenesis. If chronic hypoxia functions as an upstream driver of malignant transformation, then measurable hypoxic stress should precede or accompany early phenotypic alterations and influence the trajectory of clonal evolution. These predictions can be evaluated using established *in vivo* and *in vitro* systems in which tissue oxygenation can be experimentally manipulated and quantitatively assessed.

First, restoration or maintenance of tissue oxygenation during early stages of chronic inflammation should attenuate or delay progression from dysplasia to neoplasia. This prediction can be examined in validated models of inflammation-associated carcinogenesis, such as the azoxymethane/dextran sodium sulfate (AOM/DSS) model of colorectal cancer, where oxygenation can be experimentally modulated during defined early disease windows. Direct measurements of tissue hypoxia using established detection approaches—such as hypoxia-sensitive probes, oxygen microelectrodes, ELISA, or imaging-based modalities—combined with histologic grading and molecular profiling would allow causal relationships between oxygen availability and malignant progression to be evaluated. In parallel, longitudinal sampling in organoid systems maintained under precisely controlled oxygen conditions would enable testing of whether hypoxia precedes or closely coincides with the earliest emergence of clonally expanding populations, and whether experimental prevention of hypoxia delays these events.

Second, if hypoxia drives epigenetic remodeling prior to fixed genetic alterations, then early hypoxic exposure should induce reproducible changes in chromatin accessibility, DNA methylation patterns, and transcriptional programs before the accumulation of stable driver mutations. This can be tested using time-course experiments in genetically stable epithelial models exposed to graded hypoxic conditions, with serial whole-genome sequencing, methylome profiling, and chromatin accessibility assays to determine the temporal sequence of epigenetic and genetic alterations. Finally. Time-resolved molecular and lineage-based approaches can directly link oxygen availability to cellular reprogramming during malignant evolution. Serial single-cell transcriptomic and chromatin accessibility profiling, combined with hypoxia-responsive lineage-tracing systems, would permit identification of hypoxia-associated cell-state transitions and quantification of their contribution to dysplastic or malignant populations over time. These analyses should be paired with orthogonal assessments of tissue perfusion and oxygen tension to directly correlate physiologic oxygen availability with evolving cellular phenotypes and clonal architecture. Collectively, such experimental strategies would allow HAT to be rigorously evaluated, clarifying whether chronic hypoxia acts as an initiating driver, an early permissive factor, or a downstream selective pressure in carcinogenesis.

## Conclusion

The Hypoxic Adaptation Theory (HAT) offers an integrative framework that complements, rather than replaces, the Somatic Mutation Theory (SMT). While genetic mutations remain central to cancer biology, HAT proposes that chronic cellular hypoxia functions as a primary upstream driver that precedes and facilitates mutational accumulation. Persistent oxygen deprivation activates evolutionarily conserved survival pathways—most notably HIF stabilization, metabolic reprogramming toward glycolysis, pathological angiogenesis, and enhanced cellular plasticity. Although initially adaptive, these responses become maladaptive under sustained stress. Prolonged hypoxia imposes cumulative epigenetic, metabolic, and replicative strain, progressively reshaping chromatin architecture, altering epigenomic landscape, suppressing DNA repair fidelity, and destabilizing genomic integrity. Within this environment, phenotypic plasticity increases, cancer stem-like traits are reinforced, and selective pressures favor the emergence of resilient, therapy-resistant clones. In this view, mutations are not purely stochastic initiating events but downstream consequences of sustained microenvironmental stress and adaptive remodeling.

HAT therefore reframes carcinogenesis as the pathological endpoint of chronic hypoxic adaptation—an evolutionary survival program that, when persistently activated, drives malignant transformation. This perspective integrates genomic instability, epigenetic reprogramming, tumor microenvironment dynamics, and metabolic reorganization into unified model. By positioning hypoxia as a central organizing principle in tumor initiation and progression, HAT opens new avenues for prevention, early intervention, and therapeutic strategies aimed at correcting or reversing the underlying hypoxic stress before irreversible malignant evolution occurs.

## Future perspective

Oxygen played a critical role in the evolution and diversification (species and cell types) of mammals. Its abundance allowed organisms to harness vast amounts of energy, supporting the rise of larger and more complex animals. While oxygen enabled mammals to flourish, it also created a profound dependence—every cell requires a steady, uninterrupted supply. When oxygen source is tenuous, particularly in chronic inflammation and disease, cells experience hypoxia that severely impairs their function. Restoring oxygen is a key step in preserving cellular integrity and maintaining health.

Exercise, an anti-inflammatory dietary pattern, stress reduction, and smoking avoidance can support overall health and optimize tissue oxygen delivery. However, when circulatory function is compromised, cellular hypoxia becomes inevitable. Restoration of oxygen availability can suppress HIF signaling and reverse hypoxia-induced cellular maladaptations (Dudley and Griffioen, 2023). Accordingly, these lifestyle interventions represent effective tools for disease prevention.

Hyperbaric oxygen therapy (HBOT) increases the amount of dissolved oxygen in plasma by delivering 100% oxygen at pressure greater than atmospheric pressure (typically 2–3 atm absolute). Under normobaric conditions, hemoglobin is already ∼97% saturated, therefore, the primary physiological effect of HBOT is not increased hemoglobin saturation but a substantial rise in oxygen dissolved directly in the plasma. This markedly elevates arterial oxygen tension (paO_2_), enhances oxygen diffusion gradients, and facilitates delivery into hypoxic or poorly perfused tissues. The resulting increase in tissue oxygen partial pressure can promote physiological angiogenesis, improve mitochondrial function, and restore oxygen-dependent cellular processes. Clinical studies suggest that HBOT may improve erectile dysfunction (ED) in selected patients, particularly those with vasculogenic etiologies. Reported improvements—approaching 80%–90% in some cohorts—have been associated with enhanced microvascular perfusion of the penile bed, with angiogenic changes observed on perfusion MRI. These findings support the concept that reversing tissue hypoxia can restore endothelial function and vascular integrity (Hadanny et al., 2018). Because tumor hypoxia contributes to chemoresistance and radioresistance, strategies that increase intratumoral oxygenation may exert synergistic therapeutic effects. Hypoxic tumor regions exhibit stabilization of HIFs, which drive angiogenesis, metabolic reprogramming, stemness, and treatment resistance. By increasing oxygen availability, HBOT can transiently reduce HIF stabilization, enhance ROS generation during radiotherapy, and improve the cytotoxic efficacy of certain chemotherapeutic agents. Preclinical studies indicate that HBOT may reduce tumor hypoxia and cancer stem-like phenotypes without directly promoting tumor proliferation. However, HBOT must be used cautiously in oncology. It is contraindicated with certain chemotherapeutic agents associated with pulmonary toxicity—such as bleomycin, doxorubicin, and other anthracyclines—because elevated oxygen tensions may exacerbate oxidative lung injury and increase mortality risk. When appropriately utilized, HBOT may function as an adjuvant to radiotherapy, chemotherapy, or immunotherapy by improving intratumoral oxygenation and enhancing immune cell infiltration, though clinical evidence remains heterogeneous (Meng et al., 2025; Yuen et al., 2023).

Oxygen microbubbles (OMBs) are nano-to microscale carriers engineered to transport and deliver molecular oxygen to hypoxic tissues. Each microbubble consists of a gaseous oxygen core encapsulated within a stabilizing shell composed of lipids, polymers, proteins, or surfactants. This shell confers structural stability, prolongs circulation time, and enables controlled oxygen release. Their small size and physicochemical properties allow them to navigate compromised microvascular networks and deliver oxygen directly to regions with impaired perfusion. Preclinical animal studies demonstrate that intravenous administration of OMBs enhances tumor response when combined with radiotherapy. Improved tumor oxygenation increases radiosensitivity by restoring oxygen-dependent fixation of radiation-induced DNA damage. In murine models, co-administration of OMBs with the chemotherapeutic agent doxorubicin resulted in greater intratumoral drug accumulation compared with chemotherapy alone. This effect was associated with partial normalization of tumor vasculature, including increased vascular maturity, reduced permeability and leakage, and improved functional perfusion. Such findings suggest that transient correction of hypoxia can modulate abnormal tumor microenvironment and enhance therapeutic delivery (Ho et al., 2019). OMBs administered intravenously—and in some experimental systems, enterally (Owen et al., 2021)—represent a promising investigational platform for treating hypoxia-driven pathologies. Unlike erythrocytes, which depend on intact vascular flow for oxygen transport, OMBs may diffuse through or bypass regions of compressed, obstructed, or dysfunctional microvasculature. Once in proximity to hypoxic cells, oxygen is released and diffuses passively down its concentration gradient, restoring local tissue oxygen tension. Elevated oxygen availability can suppress HIF signaling, attenuate maladaptive angiogenic responses, and promote vascular normalization. As microvascular architecture stabilizes and perfusion improves, endogenous hemoglobin-mediated oxygen delivery may progressively resume. Beyond their therapeutic potential, OMBs provide a valuable experimental platform to interrogate the role of hypoxia in disease pathogenesis. In the context of the Hypoxic Adaptation Theory (HAT), OMBs offer a controlled method to reverse tissue hypoxia and evaluate whether correction of oxygen deficits alters epigenetic reprogramming, genomic instability, treatment resistance, or malignant progression. However, their broader clinical translation requires rigorous evaluation of pharmacokinetics, biodistribution, shell stability, oxygen-release dynamics, immunogenicity, and long-term safety.

## Limitation

The Hypoxic Adaptation Theory of cancer (HAT) is presented as a conceptual framework rather than a definitive causal model of carcinogenesis, and several limitations warrant consideration. First, much of the supporting evidence is associative. Although chronic hypoxia is consistently linked to tumor initiation, progression, and therapeutic resistance, it remains challenging to unequivocally distinguish hypoxia as a primary initiating driver of malignant transformation *versus* early permissive or reinforcing condition. Establishing temporal causality in human tissues is inherently difficult, and many observations derive from experimental or correlative clinical studies. Second, HAT does not fully account for cancers clearly initiated by strong, well defined genetic events. For example, colorectal carcinoma demonstrates relatively predictable clonal expansion driven by sequential genetic alterations. Pre-malignant conditions such as clonal hematopoiesis and monoclonal gammopathy of undetermined significance further illustrate that driver mutations can arise and expand prior to overt malignancy (Fearon and Vogelstein, 1990; Jaiswal et al., 2014; Kyle et al., 2006). In addition, several hematologic malignancies are characterized by dominating initiating genetic lesions, including the Philadelphia chromosome and PML-RARA rearrangements in acute promyelocytic leukemia (Deininger et al., 2000; de et al., 2010). While such cases may appear to challenge HAT, genetic initiation and hypoxia-driven adaptation are not mutually exclusive processes. Driver mutations may arise and clonally expand in relatively normoxic tissues, whereas hypoxia may operate in parallel or downstream as a selective pressure that shapes tumor progression, cellular plasticity, and therapeutic resistance (Semenza, 2012). Importantly, genetically defined malignancies—particularly certain hematologic cancers—may not fully represent the broader spectrum of common solid tumors, which typically emerge through more complex evolutionary trajectories involving dynamic interactions between genetic alterations and the tissue microenvironment. Third, the framework is largely synthetic, integrating existing experimental, epidemiologic, and clinical observations rather than providing direct prospective causal validation. HAT generates testable hypotheses but rigorous longitudinal, mechanistic, and interventional studies to clarify whether hypoxia is sufficient, necessary, or context-dependent in tumor initiation. Finally, therapeutic implications related to oxygen modulation must be interpreted cautiously. Oxygen delivery may exert context-dependent effects, including exacerbation of oxidative stress or unintended stimulation of tumor metabolism. Cancer evolution is inherently multi-axial, shaped by interacting genetic, epigenetic, metabolic, mechanical, vascular, and immune-mediated selective pressures beyond HIF signaling alone. Accordingly, HAT should be viewed not as a replacement for mutation-based models, but as an integrative framework emphasizing chronic hypoxic stress as a central and potentially unifying contributor to carcinogenesis (Bonnet and Walsh, 2005).

## Data Availability

The original contributions presented in the study are included in the article/supplementary material, further inquiries can be directed to the corresponding author.
